# Microbiome Modulation as a Therapeutic Approach in Chronic Skin Diseases

**DOI:** 10.3390/biomedicines9101436

**Published:** 2021-10-10

**Authors:** Karina Polak, Antal Jobbágy, Tomasz Muszyński, Kamila Wojciechowska, Aleksandra Frątczak, András Bánvölgyi, Beata Bergler-Czop, Norbert Kiss

**Affiliations:** 1 Doctoral School, Medical University of Silesia, 40-055 Katowice, Poland; m.carrine@gmail.com (K.P.); k.wojciechowska249@gmail.com (K.W.); 2 Department of Dermatology, Venereology and Dermatooncology, Semmelweis University, H-1085 Budapest, Hungary; antaljobbagy@gmail.com (A.J.); banvolgyi.andras@med.semmelweis-univ.hu (A.B.); 3 Doctoral School of Medical and Health Sciences, Jagiellonian University Medical College, 31-530 Cracow, Poland; thomasmuszynski@gmail.com; 4 Chair and Department of Dermatology, Medical University of Silesia, 40-027 Katowice, Poland; ola.fratczak89@gmail.com (A.F.); bettina2@tlen.pl (B.B.-C.)

**Keywords:** microbiome, probiotics, prebiotics, synbiotics, atopic dermatitis, psoriasis, chronic ulcers, seborrheic dermatitis, burns, acne

## Abstract

There is a growing quantity of evidence on how skin and gut microbiome composition impacts the course of various dermatological diseases. The strategies involving the modulation of bacterial composition are increasingly in the focus of research attention. The aim of the present review was to analyze the literature available in PubMed (MEDLINE) and EMBASE databases on the topic of microbiome modulation in skin diseases. The effects and possible mechanisms of action of probiotics, prebiotics and synbiotics in dermatological conditions including atopic dermatitis (AD), psoriasis, chronic ulcers, seborrheic dermatitis, burns and acne were analyzed. Due to the very limited number of studies available regarding the topic of microbiome modulation in all skin diseases except for AD, the authors decided to also include case reports and original studies concerning oral administration and topical application of the pro-, pre- and synbiotics in the final analysis. The evaluated studies mostly reported significant health benefits to the patients or show promising results in animal or ex vivo studies. However, due to a limited amount of research and unambiguous results, the topic of microbiome modulation as a therapeutic approach in skin diseases still warrants further investigation.

## 1. Introduction

In 1683, Antoni van Leuwenhoek made the first microscopic observation of bacteria colonizing the surface of the human skin [1]. Joshua Lederberg first suggested the term microbiome in 2000, meaning the collective genome of commensal, symbiotic and pathological bacteria, archaea and eukaryote of the human body [2].

The skin microbiome includes bacteria, fungi, viruses, micro-eukaryotes (mites), archaea, and phages [3]. They can be found not only on the surface of the epidermis, but also in sweat, sebaceous glands and associated hair follicles [4]. The composition of microbiome differs among different regions affected by numerous factors including age, gender, genetics, immunity, hormonal balance, sleep routine, stress, metabolic factors, hygiene and skin care routine, chemical or ultraviolet radiation exposure, physical activity, climate, environmental pollution and availability of nutrients [5]. The initial colonization of the skin depends on the delivery mode, with neonates delivered vaginally acquiring the species (*spp.*) present in the vaginal tract (e.g., *Lactobacillus, Prevotella, Sneathia*) in contrast to children delivered by Cesarean section, that acquire microbiome associated with the skin (e.g., *Staphylococcus, Corynebacterium, Cutibacterium*) [6,7]. The skin microbiome of newborns is less complex than for adults [8]. In adults, the longest assessment of the skin microbiome composition lasted for 2 years, indicating that the skin microbiome remains rather stable despite changes in the environment [9]. The most dominant group in the skin microbiome are bacteria [10]. The most dominant species are *Staphylococcus epidermidis*, *Cutibacterium acnes* and *Corynebacterium*, which overall are estimated to constitute 45–80% of the skin microbiome [5]. Considering bacteria, sebaceous and dry areas are dominated by *Cutibacterium spp*. Moist environments, with a greater humidity level, harbor mostly *Staphylococcus* and *Corynebacterium* [11]. Regarding fungi, *Malassezia spp.* are present on the whole body surface, predominant in oily sites (face, back); however, in the foot site the fungal diversity is greater [11]. The viruses identified on the human skin include *Papillomaviridae, Polyomaviridae* and *Circoviridae* families [4]. The dust mites, found in 23–100% of the population, are considered commensals; however, it is known that *Demodex* mites may be associated with blepharitis and rosacea [3]. The data on the type and role of the phages are limited, yet it was found that they can modulate the skin microbiome [12]. Skin microbiome alterations were found in the background of numerous dermatological diseases, including acne, atopic dermatitis (AD) and psoriasis, among others [13,14].

Similarly to the skin, the gut microbiome starts to shape after the delivery. Depending on dominant genera, three robust clusters of intestinal microbiome, referred to enterotypes, may be distinguished: enterotype 1 with dominant *Bacteroides*, enterotype 2 with dominant *Prevotella* and enterotype 3 with dominant *Ruminococcus* [15]. The interactions between the gut microbiome and the skin are complex and not yet fully elucidated; however, several pathways bringing light to this topic may be found [16]. Gut bacterial dysbiosis may lead to reduced short-chain fatty acid (SCFAs) production, as well as disruption of the gut barrier integrity and increased permeability, that results in bacterial translocation, activation of immune cells to produce pro-inflammatory cytokines and promotes chronic, low-grade systemic inflammation [14].

Probiotics are defined by the World Health Organisation as living microorganisms that confer a health benefit when administered in adequate amounts [17]. Administering probiotics results in the stabilization of the gut bacterial community, restoration of the bacterial microbiota “signature” in the gut, producing bacteriocins, altering microRNA (miRNAs), competing with pathogens for certain nutrients and improving the gut barrier function [18].

Antibiotics have a diverse effects on the skin and gut microbiome’s ecological balance, depending on the antibiotic class, dosage and duration [19]. Antibiotic therapies are essential in the treatment of chronic dermatological diseases including acne and rosacea [20]. They possibly reduce the propotion of pathogenic bacteria and promote the growth of potentially beneficial microorganisms [21]. However, dysbiosis of the healthy gut microbiome’s composition, induced by antibiotics, can cause and aggravate disease [22]. Moreover, antibiotic therapies have a high rate of adverse reactions and their overutilization increases the probability of developing resistance [21].

Prebiotics are substances, such as carbohydrates or fibres, that can promote the growth of beneficial bacteria. They can be defined as selectively fermented ingredients that allow specific changes in the composition as well as in the activity of the gastrointestinal microflora. Similarly to probiotics, they confer benefits upon host health. As prebiotics are typically fibers that cannot be digested by the host, they are metabolized by the gut microbiome in the colon, which results in an abundance of certain bacterial species and metabolites production, including SCFAs [4]. The combination of probiotics and prebiotics, administered simultaneously, is referred to as ‘synbiotics’, where the two agents show synergism [23].

Pre- and probiotics, modifying the gut microbiome, may be used for targeting skin health [24]. In the present paper, it was aimed to review the current knowledge concerning skin diseases, in which the supplementation of pre- or probiotics is beneficial via the modulation of the skin or gut microbiome.

## 2. Materials and Methods

**Figure 1:**
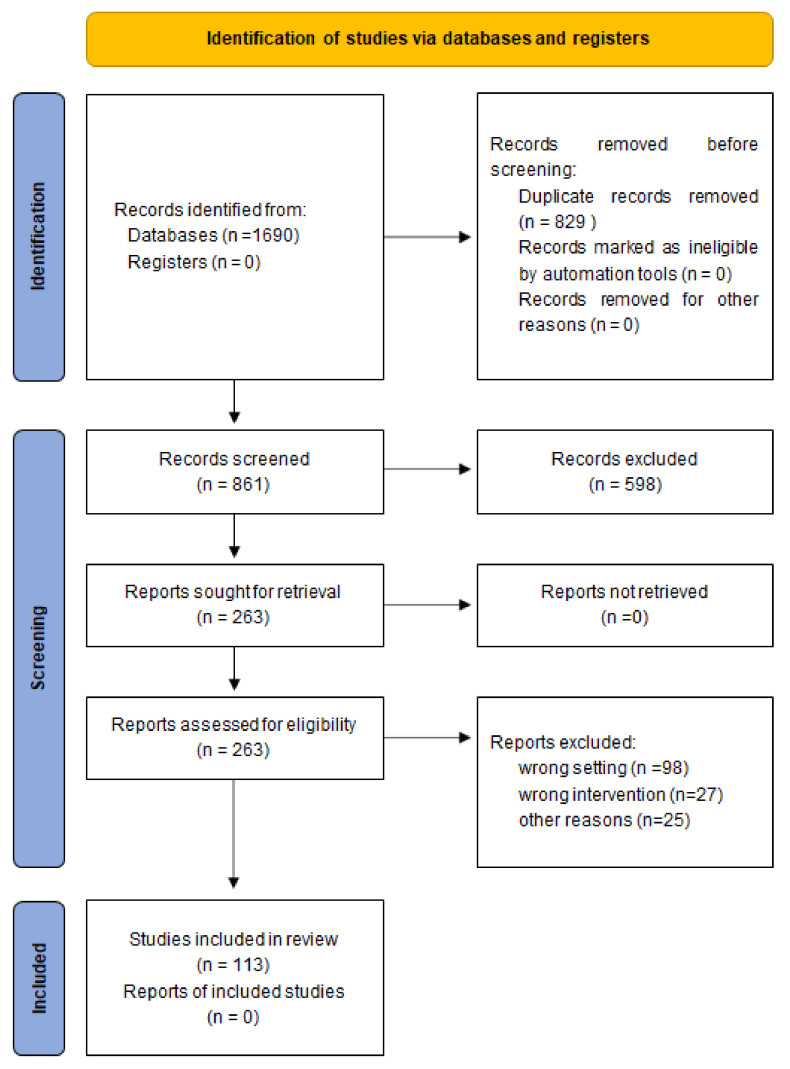
PRISMA Flow Diagram for the identification and screening of the included studies.

A search of PubMed (MEDLINE) and EMBASE databases was conducted, using a combination of keywords such as: “microbiome”; “modulation”; “prebiotic”; “probiotic”; “skin”; “skin disease” using MeSH and Emtree methods. The majority of results concerned the following diseases: AD, psoriasis, chronic wounds, SD, burns and acne. A second search in PubMed (MEDLINE) and EMBASE databases was conducted, using a combination of keywords such as: “probiotic” or “prebiotic” and the name of each of the mentioned diseases. The literature review was based on the PRISMA principles (Figure 1). Works in English published until June 2021 were included with inclusion criteria as follows: full text articles available, use of probiotics or prebiotics to treat the skin diseases. The total number of records considered into analysis was 563 on AD, 188 records on psoriasis, 628 records on ulcers, 20 records on SD, 126 records on burns and 165 records on acne. After the duplicates were removed, 470 records on AD, 101 on psoriasis, 78 on ulcers, 17 on SD, 30 on burns and 165 on acne were further analyzed. As a very limited number of studies were available on the topic of microbiome modulation with probiotics and prebiotics supplementation in all skin diseases except for AD, the authors decided to include case reports, original studies on animal or in vitro model in cases of other skin diseases. 113 articles were included in the final analysis concerning the effects of probiotics, prebiotics and synbiotics supplementation in AD, SD, psoriasis, burns, chronic ulcers and acne. We analyzed the number of patients, race, country of origin of the study population, study and control group size, study type, type of intervention and outcomes.

## 3. Results

**Atopic dermatitis.** We identified 21 original studies which investigate the influence of probiotic supplementation in pregnant women and newborns with family history of AD or allergic diseases (Table 1). Additionally, 11 studies assessed the prevention of AD with prebiotics (Table 2). 37 original studies present the treatment of AD with probiotics and prebiotics in infants (Table 3), children (Table 4) and adults (Table 5). No published data are available on the use of prebiotics in the treatment of adult patients with AD.

**Psoriasis.** Three publications concerning the administration of probiotics were found—one case report and two original studies. Four original studies investigating animal models were identified. No studies concerning prebiotics administration in psoriatic patients were found. The results are presented in Table 6.

**Chronic ulcers.** One case report and two clinical trials were found. Two studies concerning probiotics supplementation in the animal model were also identified; the authors also included two in vitro studies on probiotics application (Table 7). No published data on the topic of prebiotics supplementation in chronic ulcers was found.

**Seborrheic dermatitis.** Two clinical trials addressing oral administration of probiotics and one concerning topical administration were identified. One of the studies reported SD as a side effect of probiotic administration (Table 8).

**Burns.** 16 studies reporting the influence of pre- or probiotics on the healing of burns, the permeability of gut barrier in patients suffering from burns or the complications including sepsis were found, including seven original studies and two case reports. Both in clinical trials and in the animal model, the oral and topical administration of probiotics was investigated. One animal study also investigated the effects of local administration with daily sub-eschar injections. A single study on the prebiotics influence on the gut barrier permeability was also reported (Table 9).

**Acne.** 11 studies on the effects of probiotics and prebiotics on acne were included in the final analysis: seven clinical trials, divided into two groups with oral supplementation or topical application, and four in vitro studies. Studies concerning the use of bacterial strains not considered as probiotics (*C. acnes, S. epidermidis)* or probiotics modulating gut microbiome during antibiotic therapy were excluded from the analysis. The results are presented in Table 10.

## 4. Discussion

### 4.1. Atopic Dermatitis

AD is a common, chronic inflammatory skin disease, affecting almost 3% of adults and up to 10–20% of the child population, with an increasing prevalence. The onset usually occurs during the first year of life. AD is characterized by dry skin, pruritus and recurrent eczematous lesions. The severity of AD may be assessed by SCORAD (scoring atopic dermatitis) severity score [137]. AD is often associated with other atopic diseases: allergic rhinitis and asthma [138]. The skin and gut microbiome in adult AD patients is affected among others by maternal diet during pregnancy, the mode of delivery, antibiotics taken during pregnancy and in infancy, chronic exposure to allergens [139]. It is estimated that in over 90% of cases both lesional and non-lesional skin of the patients is colonised with *S. aureus* in AD, compared with less than 5% of healthy individuals. Moreover, in the affected areas, the abundance of *S. aureus* was associated with disease severity [140]. Increase in fungal diversity and the presence of unique anaerobic bacterial species such as *Clostridium* and *Serratia spp.* was also found on the skin of AD patients [13].

**Prevention of thedevelopment ofatopic dermatitis using probiotics**. Eight out of 21 studies reported a decreased occurrence of AD in the probiotics group. Most studies investigated *L. rhamnosus* GG (LGG). The positive impact of probiotics has been proven by Kalliomaki et al. According to the results, AD is diagnosed in 46 of 132 (35%) children aged two years, with the frequency of AD in the probiotic group (LGG) half that of the placebo group (15/64 [23%] vs. 31/68 [46%]) [25]. Wickens et al. in their study examined the *L. rhamnosus* HN001 (HN001) and HN019 influence on AD, founding the probiotic group with significantly lower cumulative prevalence of eczema and skin prick test sensitization [29]. Another study by Wickens et al. proved that mother and child intervention with HN001 probiotic supplementation was associated with a reduction in eczema and SCORAD. Note that maternal-only HN001 supplementation did not significantly reduce the prevalence of eczema in the infant by 12 months [43]. In 2006, Rautava et al. examined the use of LGG and *Bifidobacterium lactis* Bb-12 (Bb-12) in a 12-month follow-up trial. AD developed in 4/32 (13%) of the infants receiving probiotics and 8/40 (20%) of those receiving placebo [26]. Six years later, Rautava et al. confirmed the impact of daily probiotics intake (either the combination of *L. rhamnosus* LPR and *B. longum* BL999 (BL999) or the combination of *L. Paracasei* ST11 (ST11) and BL999), showing the risk of developing AD during the first 24 months of life significantly reduced in infants of mothers receiving probiotics [38]. Schmidt et al. carried out another study involving the supplementation of LGG and Bb-12, resulting in lower incidence of AD (4.2%) in the probiotic versus placebo group (11.5%) [45] Kim et al. proved that in the probiotic group the occurrence of AD was significantly reduced compared to the placebo group at 12 months of age (36.4% vs. 62.9%) [141]. Lau et al. showed that *Escherichia coli* and *Enterococcus faecalis* significantly reduced the incidence of AD development in the subgroup of high risk infants. Ten percent (15/154) of infants in the active group developed AD compared to 19% in the placebo group. This was more pronounced in the group of infants with paternal heredity for atopy (11% vs. 32%) [40]. The study of West et al. investigated the use of *L. paracasei* F-19 and found that the cumulative incidence of eczema at 13 months of age was 9/84 in the probiotic and 19/87 in the placebo groups [32].

However, 13 out of 21 trials showed that the administration of probiotics had no impact on prevalence of AD. Studies by Allen et al., Dotterud et al., Huurre et al. and Plummer et al. showed that a mix of bacterial strains was given and revealed a similar frequency of diagnosed AD both in the study and control groups. They also showed no effect of the use of probiotics in pregnant mother and infants to avoid the development of AD [30,36,41,44]. The study by Niers et al. provided interesting results in which parental-reported eczema during the first three months of life was significantly lower in the intervention group compared with placebo, 6/50 vs. 15/52. After three months, the incidence of AD was similar in both groups [33]. The use of probiotic (*L. reuteri*) in the group of pregnant women and infants was evaluated by Abrahamsson et al. Despite the cumulative incidence of AD was similar in the *L. reuteri* and the placebo groups (36% vs. 34%), IgE-associated eczema was less common in the *L. reuteri* group, although the difference was only statistically significant during the second year of life (8% vs. 20%) [27]. Conclusions from the studies by Boyle et al., Cabana et al., Kopp et al. and Ou et al. evaluating the effect of LGG on pregnant mothers showed that there was no difference between the probiotic group and placebo in the appearance of AD among the infants [31,37,39,42]. Soh et al. examined the incidence of AD in infants receiving probiotics (*B. longi* and *L. rhamnosus*). The incidence of eczema in the probiotic group was similar to that in the placebo group (22% vs. 25%). The median SCORAD at 12 months was 17.10 in the probiotic group and 11.60 in the placebo group [34]. Investigating the effects of *L. acidophilus* in their study, Taylor et al. showed no difference in the probiotic (n = 23/89; 25.8%) and placebo (n = 20/88; 22.7%) groups [28].

**Prebiotics in the prevention of atopic dermatitis**. The number of studies on prebiotics in the prevention of AD is limited and they present inconsistent results. Studies investigated only a few prebiotic compounds: combination of galacto-oligosaccharide (GOS) and fructo-oligosahccaride (FOS), acidic oligosaccharides, polydextrose (PDX), different content of lactose, oligofructose plus inulin. Among the nine studies included in this review, five have shown the positive effect of prebiotics in the prevention of the development of eczema. The rest of the studies showed no significant differences in group of infants fed with or without prebiotics.

Positive effects of the administration of prebiotics has been shown by Ziegler et al., who investigated the administration of a GOS and PDX mix and found a statistical difference in the occurrence of eczema (prebiotics vs. control: 18 vs. 7%) [47]. The same combination was used by Pontes et al. in their study, who reported a lower number of allergic diseases including AD in the analyzed group receiving prebiotics [53]. Three trials investigating the relationship between GOS supplementation and preventing eczema shown a decreased risk of developing AD [46,48,49]. However, Grüber et al. found that a formula containing a mixture of neutral oligosaccharides can be also effective in prevention of AD [50]. Wopereis et al. also presented a beneficial impact in the prevention of AD and modulation of gut microbiota by using a partially hydrolyzed formula containing short-chain GOS and long-chain FOS and pectin-derived acidic oligosaccharides [56]. No differences between prebiotic groups and control groups have been found in four studies. Two of them investigated a mix of FOS and GOS [51,52]. Ranucci et al. used a mixture of GOS and PDX in their trial. There were no significant differences in the cumulative incidence, intensity and duration of AD among the investigated groups of patients [55]. A study by Boyle et al. on prebiotic containing FOS has shown that prebiotics did not prevent AD in high-risk infants during the first 12 months of life [54].

**Role of probiotics and prebioticsin AD treatment.** 20 of 27 studies on probiotics revealed improving SCORAD in AD patients compared to placebo. One of the first studies on probiotic treatment in AD, that found using probiotics may have positive impact on the course of AD, was the study by Isolauri et al. The aim of their study was to evaluate the effects of probiotics use with Bb-12 or LGG on infants with AD. The results showed that by using probiotics, the skin condition improves. SCORAD decreased in the Bb-12 group to 0, and in the LGG group to 1 versus the SCORAD of 13.4 in the placebo group [58]. Drago et al. evaluated the influence of *S. thermophilus* ST10 and tara gum on the SCORAD score. The score decreased significantly in the probiotic group after one month and the index was significantly lower in the probiotic group than in the placebo group [92]. Ivakhnenko et al. evaluated both the use of Bb-12 and *Streptococcus thermophilus* for 4 weeks. The results showed significant improvement of SCORAD in the probiotic group compared to blacebo [73]. Wu et al. proofed that the SCORAD index declined from baseline after two months in the LGG group [76]. Brouwer, Folster-Holst and Kirjavainen showed similar effects of this LGG bacteria in AD patients [59,63,64]. Studies which resulted in a significant decrease in the SCORAD index in AD patients by using probiotics containing single strains or combined bacterial strains, including *L. salivarus* LS01, were also those conducted by Drago et al. [89] and Iemoli et al., [90] *L. acidofilus* DDS-1—by Gerasimov et al. [80], *L. plantarum*—by *Han* et al. [82], *L. fermentum*—by Weston et al. [61] and *L. sakei*—by Woo et al. [81] Yang et al. randomly assigned their patients to the probiotic-receiving groups (*L. casei, L. rhamnosus, L. plantarum*, and *B. lactis*) or placebo groups for six weeks. The result of their trial was a significant clinical improvement in the skin condition among the probiotic groups [85]. Two of the studies also proved a positive impact on the SCORAD score by using probiotics with *B. breve*. Taniuchi et al. and Yoshida et al. showed a significant improvement in skin conditions during the study in the probiotic group [62,88]. Three studies assessing the impact of using bacteria mix in probiotic groups revealed a significant improvement in SCORAD scores [84,86,87].

Seven out of 27 studies in children showed no significant differences in SCORAD scores between the probiotic and placebo groups after treatment. Lin et al. proved that the SCORAD index was not significantly reduced in the *B. bifidum* group versus controls [74]. Grüber et al. suggested that AD improved after four weeks of supplementation (LGG vs. placebo); however, the difference was not significant [65]. Similar results were obtained by Viljanen et al.: the SCORAD score decreased by 65%, but with no statistically significant differences between treatment groups [60]. Sistek et al. evaluated the role of *L. rhamnosus* and *B. lactic*. Their findings stated that there is no significant difference between probiotic and placebo groups [78]. Rosenfeldt et al. examined probiotic *Lactobacillus* strains (lyophilized *L. rhamnosus* 19070-2 and *L. reuteri* DSM 122460) in combination for six weeks in 1- to 13-year-old children with AD. The total SCORAD index in this trial did not change significantly [77]. The results of the study by Gøbel et al. study on *L. acidophilus* and *B. lactis* Bi-07 (Bi-07) were that there was no benefit for the probiotics on the severity of AD. However, a post hoc analysis showed a significant reduction in severity of AD in the Bi-07 group and possible positive effects of this probiotic strain could be of further interest [68]. Gore et al. in their trial compared the effects of using *B. lactis* and *L. paracasei*. No significant differences were observed between the groups after 12-week treatment-period [71].

Five out of 36 studies did not estimate the SCORAD score, but evaluated other factors, such as puritus. Matsumo et al. in their study found that *Bifidobacterium animalis* subsp. *lactis* LKM512 may reduce pruritus by increasing expression of metabolite kynurenic acid [91]. The results obtained by Majama et al. suggest that probiotic bacteria may improve endogenous barrier mechanisms in patients with AD and those with food allergies by decreasing intestinal inflammation and may be useful in AD treatment [57]. Studies by Flinterman et al. [66], as well as by Guo et al., suggested that in vitro IgE production is decreased in the probiotic group compared to placebo [75]. Nermes et al. found that the levels of IgA and IgM-sectreting cells decreased significantly in the probiotic group compared to placebo. The baseline-adjusted ratios for treated to untreated patients after one month were 0.59 for IgA- and 0.53 for IgM-secreting cells [69].

**Synbiotics.** Five publications on the use of synbiotics were found; however, only one of them by Farid et al. reported a significant reduction of the SCORAD score [70]. Passeron et al. compared the effects of probiotics (*L. rhamnosus* Lcr35) and synbiotics in children over two years old. The study showed no statistical differences regarding SCORAD scores between the two groups [79]. Shafiei et al. showed that there is no significant difference in the mean decrease of total SCORAD between placebo (22.3) and synbiotic groups (24.2) [72]. A significantly greater SCORAD score improvement was found in the symbiotic group of infants with IgE-associated AD by van der Aa et al. [67] Wu et al. also found that a combination of *L. salivarius* and FOS resulted in lower SCORAD in a comparison with the control [83].

The role of microbiome composition in allergic diseases is well-known, with lower biodiversity found as a factor inducing their development. Modulating the microbiome with probiotics balances the gut microflora, protects the function of intestinal barrier and lowers the level of pro-inflammatory cytokines produced. Probiotics also influence Toll-like receptors, which play an important role in T-cell differentiation and the development of allergic reactions. As skin colonization with *S.* *aureus* plays an important role in AD, a promising new perspective of displacing it with more desirable species is also considered [93].

### 4.2. Psoriasis

Psoriasis is a common inflammatory disease that affects around 2–3% of the population [142]. It manifests with papulosquamous skin lesions with variable distribution and severity [143]. The pathogenesis of the disease is not yet fully elucidated. However, it is known that genetic, immunological and environmental factors may act as triggering factors, making the keratinocytes start secreting pro-inflammatory cytokines [14]. In the skin lesions, increased abundance of *Streptococcus spp., Corynebacterium spp., Cutibacterium spp., Staphylococcus spp., Finegoldia spp.* and *Neisseria spp*. can be found. The biodiversity of microbiota is generally decreased in moderate-to-severe psoriatic patients in contrary to mild psoriatic patients [5].

Based on the reported alterations in gut microbiome, attempts were made to use probiotics and prebiotics in the treatment of psoriasis. Two original studies, one case report and four mice studies were published. In the case report described by Vijayashankar and Raghunath, a supplementation with *L. sporogenes* for 15 days allieviated the symptoms accompanying the sudden onset of generalised pustular psoriasis in a 47-year-old female [94]. Groeger et al. demonstrated a significant decrease in serum CRP, TNF-α levels in psoriatic patients administered with *B. infantis* 35264 for 8 weeks [95]. In the study by Navarro-Lopez et al., supplementation with *B. longum* CECT 7347, * B. lactis* CECT 8145 and *L. rhamnosus* CECT 8361 for 12 weeks resulted in a significant reduction in PASI scores [96]. In three out of four mice studies, the probiotics were administered orally, while in one study it was administered topically. In all studies, the psoriasis-like skin inflammation was induced by topical appliaction of imiquimod. Chen et al. found that in administering *L. pentosus* GMNL-77, both for five or seven days, causes a reduction in erythaematous scaling lesions, decreases TNF-α, IL-6, IL-23, IL-17A/F and IL-22 levels in the skin, decreases spleen weight and reduces the number of IL-17- and IL-22-producing CD4+ T cells in the spleen [97]. In the study by Lu et al., seven different groups of six mice each were given different strains of probiotics. *B. adolescentis* CCFM667, *B. breve* CCFM1078, *Lacticaseibacillus paracasei* CCFM1074, and *Limosilactobacillus reuteri* CCFM1132 ameliorated psoriasis-like pathological characteristics and suppressed the release of IL-23/T helper cell 17 (Th17) axis-related inflammatory cytokines. On the contrary, *B. animalis* CCFM1148, *L. paracasei* CCFM1147 and *L. reuteri* CCFM1040 neither alleviated the pathological characteristics nor reduced the levels of inflammatory cytokines [99]. Ogawa et al. showed that administering *Leuconostoc mesenteroides* NTM048 to imiquimod-induced mice suppressed erythema, scaling, upregulated IL-17 production, increased the levels of plasma deoxycholic acid and altered the faecal microbiota composition. Changes in the gut microbiome were indicated by the increased abundance of *Akkermansia* and a decreased abundance of *Staphylococcus* and *Streptococcus* [100]. The only study concerning a topical application of probiotics was conducted by Rather et al. Application of ethanol extract (SEL001) isolated from *L. sakei* proBio-65 resulted in an inhibition of the imiquimod-induced changes in the skin, as well as decreased IL-19, IL-17A and IL-23 levels [98]. It was shown that the gut microbiome plays an important role in the pathogenesis of psoriasis—patients suffering from this disease present with an increased amount of *Bacteroidetes* and decreased levels of *Firmicutes, Proteobacteria* and *Actinobacteria,* probably altering the intestinal barrier integrity, T-cell response and population-type balance, chemotaxis along with carbohydrate, cobalamin, and iron metabolism [144].

### 4.3. Chronic Ulcers

The use of probiotics as a novel treatment for diabetic foot ulcers (DFU) was first published in 2014. It was suggested that the application of probiotic agents would enable the healing of diabetic ulcers and would prevent diabetic foot infections by activating Toll-like receptors and producing β-defensins, which stimulate skin immune functions [145]. Mohseni et al. investigated the advantages of probiotics in patients with DFU. After the 12-week intervention of probiotic supplementation (*L. acidophilus, L. casei, L. fermentum, B. bifidum*), it had beneficial effects on the DFU size. It also decreased the serum total cholesterol and CRP and increased plasma nitric oxide (NO) and total plazma antioxidant capacity [102].

Most research was carried out using in vitro models, e.g., the effectiveness of a probiotic based on *L. rhamnosus* and *L. paracasei* strains in a 1:1 ratio against microorganisms previously isolated from chronic ulcerative lesions. Following the administration of probiotics, the growth of bacteria, compared to the control, was lower in the case of such bacteria as *P. aeruginosa, C. striatum, A. baumanii, S. aureus, P. mirabilis* in 75%, in the case of *Candida parapsilosis* in 93.75%, while in the case of *E. faecalis* 18.75%, and 50% for the mixed flora of the mentioned pathogens. The ability to co-aggregate all pathogens that could prevent adhesion and invasion was also shown [108]. Kusumaningsih et al. investigated the differences in the number of fibroblast cells and blood vessels after the administration of the probiotic *L. casei shirota* topically and systemically during the onset of the healing of traumatic ulcers in Wistar rats. The number of fibroblasts and new blood vessels were significantly higher in the two intervention groups in a comparison with the control group [106]. A further study investigated wounded New Zealand rabbits infected with *S. aureus* and treated with *L. fermentum,* which secrets gaseous NO. The day after the procedure, treatment with the patch with a probiotic agent started and lasted for 21 or 20 days. Morphometric analysis of the ulcer healing revealed that it was significantly accelerated with this treatment method in both infected and uninfected ischemic wounds [104]. Stefia et al. compared the effects of two different strains of *Faecalibacterium prausnitzii* (SPA and SPAH) for immune cell activity and wound healing in mice. They found that the presence of these strains in the gut exhibited significantly higher patterns of reepithelialization compared to controls by inhibiting *NF-kβ* activation. It resulted in decreased wound proinflammatory cytokine expression and induced myofibroblast and collagen transitions [105]. In another model, *L. reuteri* was transformed with a plasmid containing the genetic material of the C-X-C Motif Chemokine Ligand 12 chemokine involved in accelerated wound healing. Additionally, the lactic acid produced by the probiotic bacterium lowered the pH and increased the bioavailability of the chemokine. This strain was applied to wounds in mice, accelerating ulcer healing, epithelialization, and wound closure [107].

The most relevant original work was published in 2010. *L. plantarum* was used in the treatment of chronic leg ulcers. The probiotic was applied to ulcers in 14 patients with diabetes and 20 non-diabetic patients. After 30 days of follow-up, 90% of the extent of ulceration had resolved in 43% of diabetic patients and 50% of non-diabetic patients. A decrease in CFU of *S. aureus, S. epidermidis* and *P. aeruginosa* was also noted. It was found that probiotics disrupt biofilm, regulate IL-8 levels and modulate the immune system [101]. In addition, Venosi et al. reported a case of an old woman who was successfully treated with a topical administration of probiotics for an ischemic and infected (*K. pneumoniae, E. faecalis* and *P. mirabilis*) chronic wound. The patient received a mixture of probiotics (*L. plantarum, L. acidophilus* and *S. thermophiles*) three times/week [103].

### 4.4. Seborrheic Dermatitis

Seborrheic dermatitis (SD) characterized by erythematous, scaling plaques on the the face, chest and scalp [146,147]. It is assumed that the underlying cause of the disease is the excessive activity of sebaceous glands and concomitant infection with *Malassezia spp.* [146]. Research indicates an increased number of *Malassezia* strains in the seborrheic area and a satisfactory therapeutic effect of antifungal formulas [147]. Currently, it seems that SD is the result of the skin’s response to free fatty acids produced by *M. furfur*, which elicit an inflammatory response from keratinocytes [148,149]. *M. furfur* also possesses the ability to produce metabolites, which stimulate the aryl hydrocarbon receptor and thus may modulate the function of antigen-presenting cells [150].

There are limited data on the effects of probiotics and the modulation of the cutaneous microbiome on the course of SD. The use of superficial *Vitreoscilla filiformis* preparation in a double-blind study involving 60 patients with SD resulted in a reduction of itching, erythema and scaling. At the cellular and subcellular level, the lysate of these bacteria resulted in an increase in the activity of IL-10 produced by dendritic cells of the skin and an increase in the activity of regulatory T lymphocytes [109]. Another study involving the oral administration of ST11 demonstrated a significant reduction in symptoms, which at the subcellular level was also accompanied by a shift in immune activity consisting, as before, in an increase in IL-10 production [110]. These examples confirm the possible benefits of using both forms of probiotics in the group of patients with SD.

### 4.5. Burns

The analysis of studies conducted both in animal models and in clinical trials, mostly showed at aleast partial positive effect of the use of probiotics on the healing of infected wounds by inhibiting microbiome growth, microfilm formation and interbacterial communication [151].

Among the various used bacterial strains, the most evidence exists for *L. plantarum*. Peral et al. established the effectiveness of *L. plantarum* probiotic treatment with a topical application in human patients. *L. plantarum* would compete with bacterial pathogens and would be able to promote tissue repair [111]. El-Ghazzey et al. studied the effect of *L. fermentum* and *L. delbruekii* treatment in pediatric post-burn patients. They conclude that probiotic administration is safe to use and improves wound healing [115]. In a case report, oral application of *L. casei* resulted in the appearance of multi-drug sensitive *P. aeruginosa* instead of an extremely drug-resistant strain [113]. Perdanakusuma et al. demonstrated that *B. infantis* 35624 single-strain probiotics were more effective compared to *Lactobacillus reuteri protectis* in altering intestinal immunity [116].

Valdez et al. has been shown in adult inbred BALB/c mice that *L. plantarum* and/or its by-products could be a potential therapeutic agent for *P. aeruginosa* burn infections [120]. Brachkova et al. found that the application of calcium alginate films containing *L. plantarum* reduced *P. aeruginosa* in a rat model of burns [121]. Argenta et al. proved in mice that probiotic therapy (*L. plantarum*) suppressed the induction of TNF-α, IL-6 and IL-10 in liver and inhibited the accumulation of the pathogen in remote organs [122]. Satish et al. *L. plantarum* as a therapeutic agent alleivates burn wound infection and scaring after burn injury in rabbits [123]. Sürmeli et al. demonstrated that *L. plantarum* has a protective role in non-infected burn wounds against meticillin-resistant *Staphylococcus aureus* (MRSA). Additionally, the therapeutic effect of *L. plantarum* was not shown in MRSA infection [124]. Herek et al. investigated that the *Saccharomyces boulardii* could effectively decrease the incidence of antibiotic-induced bacterial translocation in burned rats [118].

Khan et al. demonstrated the importance of the method of probiotic application in a thermal burn mouse model. The use of the bioskeleton compared to traditional forms of probiotic application resulted in acceleration of epithelialization, collagen production and formation of hair follicles, as well as an inhibiton on the growth of pathogenic bacteria, reducing infection and accelerating wound healing [125]. As a result of burns due to systemic stress, the intestinal barrier is significantly impaired, resulting in inflammation and oxidative stress, leading to the destruction of the intestinal barrier and abnormal intestinal function. The studies on the animal model of burns show that the application of glutamine and probiotics reduced the apoptosis of the intestinal epithelial cells [119].

Fleming et al. performed a retrospective study in connection with preventing potential antibiotic-associated *C. difficile* colitis by giving probiotics to burned patients in a critical condition. Otherwise, they found no significant difference in *C. difficile* infection between the control group and the intervention group [117]. Olguin et al. proposed that the regular intake of prebiotics might help to increase the gastrointestinal permeability in burn patients. Following the application of oligofructose (OF), they found no difference between the control and OF groups [126].

Due to the damaged intestinal barrier and the impaired immune system function caused by burns, there is a potential risk that probiotic bacteria may translocate and ultimately result in infection. Mayes et al. demonstrated the efficacy and safety of probiotics in the pediatric population hospitalized due to skin burns [114]. However, there are known cases of severe infections and probiotic-induced sepsis in critically ill people [112].

### 4.6. Acne

Acne is a chronic skin disease, affecting the pilosebaceous units, with multifactorial pathogenesis including hormonal influence, the immunological state of the host, diet, deregulation of insulin-like growth factor, excessive sebum production and FoxO1 deficiency [152,153]. Considering the pathogenesis of acne, *Cutibacterium acnes* has been implicated as an important pathogenic factor. Fitz-Gibbon et al. compared the *Cutibacterium* strains in patients suffering from acne and healthy individuals, finding remarkable differences [154]. More and more evidence suggests that dysbiosis on the phylotype/strain level leading to a diversity loss is also a major factor in the pathogenesis of acne [155]. The role of the gut microbiome in acne is also raised, as a study conducted in 2018 showed that patients with acne present with lower gut microbiota diversity (abundance of *Firmicutes, Clostridium, Clostridiales, Lachnospiraceae, Ruminococcaceae* increased *Bacteroides* levels) [156].

A limited number of studies concerning probiotics and prebiotics use in acne is available. Yet, it is known that the beneficial components of the microflora may ameliorate skin lesions via the suppression of the Treg cell population. In addition, the suppression of B and Th cells due to the modulation of inflammatory cytokine production along with increasing IgA and butyrate secretion may also have an important effect [144]. A clinical trial investigating oral supplementation of *L. rhamnosus* SP1 (LSP1) had been reported to bring health benefits to the patients such as LSP1 normalized skin expression of genes involved in insulin signalling and an improvement in the appearance of adult acne [127]. However, a mix of *B.* *lactis* W51, *B. lactis* W52, *L. acidophilus* W55, *L. casei* W56, *L. salivarius* W57, and *L. lactis* W58 was reported to be a trigger for elevated IL-10 serum levels [129]. The results concerning oral prebiotics supplementation remain more consistent, as both lactoferrin as well as GOS and FOS were associated with positive effects [35,128]. Topical application of probiotic-enriched formulas also seem to have a promise: all of the analyzed studies involving the use of *E. faecalis* SL-5 [130], *Nitrosomonas eutropha* [131] or *L. acidophilus* showed improvement in the skin condition. *L. acidophilus* was also reported to decrease the population of *C. acnes* [132]. These findings were also confirmed in in vitro studies: Al-Ghazzewi et al. showed that probiotic bacteria and konjac glucomannan hydrolysates inhibit *C. acnes* growth [133]. Similar effects were reported by Kang et al., who investigated the properties of *L. reuteri* on the proliferation of *C. acnes* and *S. epidermidis* [134]. *Bifidobacterium spp*. [135]. as well as two *S. salivarius* strains and one *L. plantarum* strain, were also reported to show antimicrobal activity in in vitro studies against *C. acnes* and other pathogens [136].

### 4.7. Limitations

The present review focuses on a subject that is relatively new and is still not investigated in full detail. One of the major limitations is the small number of publications reporting clinical studies, especially multi-center, double-blinded, placebo-controlled clinical trials. The number of patients in the presented studies were usually low and many studies involved animal models, which cannot be extrapolated to humans. Since the exact pattern composition of a “healthy microbiome” is impossible to establish, there are no objective measures to investigate a universal model. The bacteria used in different studies presented various genera and properties; moreover, they were derived from different sources, often with no exact information on the method of production, storage and other properties. Moreover, the skin diseases presented in the paper were chosen based on their duration and the number of studies available; however, single studies show that the microbiome modulation, e.g., via a fecal microbiota transplant, may be also effective in the treatment of other dermatological conditions, for example in alopecia areata [157].

## 5. Conclusions

It can be stated that the microbiome plays an important role in dermatological diseases, at the same time as being an attractive target of therapeutic interaction. This may contribute to the promotion of beneficial (from the point of view of inflammation) activation of the immune system, a reduction of the inflammatory state and, above all, could constitute a physical barrier to the colonization of the skin by pathogenic bacteria. The perspective of treating skin diseases with microbiome modulation via oral and topical probiotics, prebiotics or synbiotics are becoming a part of reality.

There is a growing number of studies into the beneficial effects of probiotics in patients with atopic diseases. It is estimated that the oral application of probiotics or prebiotics during delivery or in the first months of life could delay or alleviate the appearance of AD in infants. From another point of view, probiotics could have the potential to reduce the SCORAD index as a treatment method. On the basis of the available evidence, a recommendation on probiotic intake in order to avoid AD cannot be currently made. Administering probiotics may influence the composition of the gut microbiome, which is more and more often considered to be a factor in the development of psoriasis. The suspected efficacy of probiotics in alleviating the course of psoriasis may be connected to lowering the levels of plasma pro-inflammatory cytokines. Since the data and the amount of research on this topic are limited, it still requires new, randomized, placebo-controlled trials, which would gain an insight into the pathogenesis and novel strategies of psoriasis treatment. There are very limited data available at the moment in the context of chronic ulcers. The positive effects of probiotics were shown mainly in studies focusing on ulcers resulting from diabetes complications. Probiotics may prevent or reduce the infection of burned wounds. Most research has focused on the *L. plantarum* and has showed at least a partial positive effect of the use of probiotics on the healing of infected wounds by inhibiting pathogen growth, microfilm formation and interbacterial communication. Concerning SD and acne, the very limited available data on probiotic administration have showed inconsistent results.

The studies have shown that probiotics and prebiotics both administered orally or applied topically may have a positive influence on the course of skin diseases. Despite the continuous increase in promising data on the effectiveness of the use of probiotics and prebiotics, further clinical trials are needed to assess the efficacy and long-term safety profile of probiotics and prebiotics in the treatment of patients with dermatological diseases.

## Figures and Tables

**Table 1:** Prebiotics in the prevention of AD.

Number (No.) of Study	Author	Patient Population (Number)	Type of Study	Intervention	Results
1	Kalliomaki et al.—2007 [25]	Pregnant women (n = 159) with a family histors of AD, continuing after delivery and their children (n = 132)	Double-blind, randomised placebo-controlled trial	Participants received two capsules of placebo (n = 95) or LGG (n = 64) daily for 2–4 weeks before expected delivery	The frequency of AD was significantly reduced
2	Rautava et al.—2006 [26]	Infants with 6 months of exclusive breast-feeding (n = 38)	Double-blind placebo-controlled study	Infant formula supplemented with either LGG and Bb-12 (n = 38) or placebo (microcrystalline cellulose) (n = 43) daily until the age of 12 months	Supplementation of probiotics increased protective cow’s milk-specific IgA responses. 13% of the infants receiving probiotics and 20% of those receiving placebo manifested with AD, cow’s milk allergy was confirmed in none of the infants receiving probiotics and in 8% of the infants receiving placebo
3	Abrahamsson et al.—2007 [27]	Pregnant women with a family history of at least 1 allergic disease (n = 188) and then their infants (n = 188)	Prospective, double-blind, placebo-controlled, multicenter trial	The mothers were taking *L. reuteri* (n = 95) or placebo (n = 93) 4 weeks before term and continued daily until delivery, after birth, the baby continued with the same product up to 12 months of age	The cumulative incidence of AD was similar in the probiotic and the placebo groups (36% vs. 34%)
4	Taylor et al.—2007 [28]	Infants with atopic mother (n = 178)	Randomized, double-blind, placebo-controlled	Newborns of women with allergy received either *L. acidophilus* (n = 89) or placebo (n = 89) daily for the first 6 months of life	Not reduction in the risk of AD and increased allergen sensitization
5	Wickens et al.—2008 [29]	Pregnant women (n = 474) and their infants (n = 474)	Double-blind randomized placebo-controlled trial	Daily supplementation with either HN001 (n = 157) or HN019 (n = 158) or placebo (n = 159) from 35 weeks gestation until birth, continuing to 6 months after birth in mothers if breastfeeding, and from birth till 2 years in all infants	Prevention of the development of AD
6	Huurre et al.—2008 [30]	Pregnant women (n = 140) and infants (n = 138)	Placebo-controlled prospective intervention study	Oral administration of LGG and Bb-12 each day (n = 72/70), or placebo (microcrystalline cellulose and dextrose anhydrate) (n = 68). Atopic sensitization was at the age of 6 and 12 months and in mothers at third trimester of pregnancy	There was no difference between infant sensitization in the probiotic and the placebo group
7	Kopp et al.—2008 [31]	Pregnant women (n = 105) with a family history of at least one allergic disease and their children (n = 96)	Double-blind, placebo-controlled prospective trial	Administration of either the probiotic LGG (n = 54) twice daily or placebo (n = 51) 4–6 weeks before expected delivery, followed by a postnatal period of 6 months	After a 2 year follow-up, administration of probiotic did not reduce the incidence nor altered the severity of AD
8	West et al.—2009 [32]	Healthy infants with birth weight >2500 g who were vaginally delivered (n = 89)	Double-blind, placebo-controlled randomized intervention trial	Daily intake of cereals supplemented with LF19 (n = 89) or identical cereals without LF19 supplementation (n = 90) from 4 to 13 months of age	Decreased cumulative incidence of AD
9	Niers et al.—2009 [33]	Pregnant women (156) and then their infants with a positive family history of allergic disease (n = 156)	Double-blind, randomized, placebo-controlled trial	Probiotic bacteria were prenatally administered to pregnant mothers (n = 78) during the last 6 weeks of pregnancy and postnatally for 12 months to their infants (n = 78); the intervention group received once daily *B. bifidum* W23, *B. lactis* W52, and *L. lactis* W58) in a freeze dried powder	Decreased incidence of AD
10	Soh et al.—2009 [34]	Infants with a positive family history of allergic disease (n = 253)	Double-blind, placebo-controlled randomized clinical trial	Infants (n = 127) received commercially available cow’s milk formula with probiotic supplementation of BL999 and *L. rhamnosus* daily for the first 6 months. Infants in the control group (n = 126) received milk without probiotics	No effect on the prevention of AD or allergen sensitization
11	Kim et al.—2010 [35]	Pregnant women with a family history of allergic diseases (n = 112), continuing after delivery and their infants (n = 68)	Double-blind, randomized, placebo-controlled trial	Pregnant woman received supplement of *B. bifidum* BGN4, *B. lactis* AD011 and *L. acidophilus* AD03 (n = 33) or placebo (n = 35), starting at 4–8 weeks before delivery and continuing until 6 months after delivery. Infants were exclusively breastfed during the first 3 months, and were fed with breastmilk or cow’s milk formula from 4 to 6 months of age	The prevalence of AD in the first year of life was significantly lower in the probiotic group.
12	Dotterud et al.—2010 [36]	Pregnant women (n = 415) and their infants (n = 278)	Randomized, double-blind trial	Pregnant women received probiotic milk (n = 138) or placebo (n = 140) from 36 weeks of gestation to 3 months postnatally during breastfeeding	Decreased cumulative incidenceof AD.
13	Boyle et al.—2011 [37]	Pregnant women (n = 250), their partner or a previous child was affected by allergic disease including asthma, eczema, food allergy or allergic rhinitis	Randomized controlled trial	Participants were allocated to take probiotic treatment with LGG (n = 125) or maltodextrin placebo (n = 125) each morning from 36 weeks gestation until delivery. Infants were assessed during their first year for eczema or allergic sensitization	Prenatal treatment was not associated with reduced risk of eczema or IgE-associated eczema but decreased breast milk soluble CD14 and IgA levels
14	Rautava et al.—2012 [38]	Pregnant women with atopic sensitization (n = 241) and their infants (n = 205)	Double-blind, randomized, placebo-controlled trial	Pregnant women received a dietary food supplement with the combination of LPR and BL999 (n = 81) or the combination of ST11 and BL999 (n = 82) or placebo (78)	Administration of specific probiotics is a safe and effective way in reducing the risk of AD
15	Ou et al.—2012 [39]	Pregnant women with atopic diseases determined by history, total immunoglobulin (Ig)E > 100 kU/L, and/or positive specific IgE (n = 191)	Prospective, double-blind, placebo-controlled clinical trial	Pregnant woman receive either LGG ATCC 53103 (n = 95) or placebo (n = 96) from the second trimester of pregnancy	Reduced severity of maternal allergic disease
16	Lau et al.—2012 [40]	Infants with at least single heredity for atopy (n = 606)	Randomized, placebo-controlled trial	From week 5 until the end of month 7, infants were treated orally with bacterial lysate containing heat-killed gram-negative *E. coli* and gram-positive *E. faecalis* (n = 303) or placebo (n = 303)	Prevention of the development of AD
17	Allen et al.—2014 [41]	Pregnant women (n = 454) and then their infants with a positive family history of allergic disease (n = 454)	Randomised, double-blind, placebo-controlled, parallel group trial	Women from 36 weeks gestation and their infants to age 6 months received daily either the probiotic (*L. salivarius* CUL61, *L. paracasei* CUL08, *B. animalis* subsp. *lactis* CUL34 *and B. bifidum* CUL20) (n = 220) or placebo (n = 234)	Cumulative frequency of AD at 2 years of age was similar between the two groups
18	Cabana et al.—2017 [42]	Infants (n = 184)	Randomized, double-blind controlled trial	The intervention group received a daily capsule of LGG and inulin for the first 6 months of life (n = 92); the control group received inulin (n = 92)	At 5 years of age, the cumulative incidence of asthma was significantly higher in the control group (17.4%) than in the intervetion group (9.7%)
19	Wickens et al.—2018 [43]	Pregnant women, continuing after giving birth. The patient or her partner had a history of atopic disease (n = 473)	2-centre, parallel double-blind, randomized placebo-controlled trial	HN001 (n = 157), HN019 (n = 158) or placebo (n = 159) was taken daily by mothers from 14-16 weeks of gestation until 6 months post-partum. Their infants were also given the the same capsule daily from birth until the age of 2 years	Prevention of the development of AD and atopic sensitization
20	Plummer et al.—2019 [44]	Preterm infants, born <32 gestational week and weighing <1500 g (n = 281)	Multi-center, double-blind, placebo-controlled randomized trial	Infants in the inetrvention group (n = 127) received a probiotic combination *B. infantis*, *Str. thermophilus*, and *B. lactis* once daily (in a maltodextrin base powder) and the placebo group (n = 154) received maltodextrin	No effect on the incidence of allergic diseases or atopic sensitization
21	Schmidt et al.—2019 [45]	Infants with birthweight >2500 g, gestational age >36 weeks (n = 144)	Double-blind, placebo-controlled intervention trial	The intervention group (n = 144) received sachets of maltodextrin supplemented with LGG and Bb-12, and the placebo group (146) received maltodextrin only	A significantly lower incidence of AD in the probiotic group

Abbreviations: AD, atopic dermatitis; *B., Bifidobacterium*; Bb-12, *Bifidobacterium lactis* Bb-12; BL999, *Bifidobacterium longum* BL999; *E. coli, Escherichia coli; E. faecalis, Enterococcus faecalis*; IgE, immunglobulin E; IgA, immunglobulin A; *L., Lactobacillus acidophilus*; LF19, *Lactobacillus paracasei* F19; LGG, *Lactobacillus rhamnosus* GG; HN001, *Lactobacillus rhamnosus* HN001; HN019, *Bifidobacterium animalis* subsp. *lactis* strain HN019; *Str., Streptococcus*; ST11, *Lactobacillus paracasei* ST11; No, number.

**Table 2:** Prebiotics in the prevention of AD.

No. of Study	Author	Patient Population (Number)	Type of Study	Intervention	Results
1	Moro et al.—2006 [46]	Infants at risk for atopy (n = 259)	Prospective, double-blind, randomised, placebo controlled trial	Participants received either hydrolysed cows’ milk with GOS/FOS in the prebiotic group (n = 102) or maltodexitrin in the control group (n = 104)	Development of AD was significantly more frequent in the control group
2	Ziegler et al.—2007 [47]	Healthy infants (n = 226)	Double-blind, randomized, controlled, parallel-group, prospective trial	Participants were divided into 3 different formula groups: control group-PDX (n = 76), PG4 group- PDX+GOS (n = 74), PDL8 group-PDX, GOS, LOS (76). Formula was fed for 120 days	No differences among the groups in growth rate
3	Arslanoglu et al.—2008, 2012 [48,49]	Healthy infants with a parental history of atopy (n = 134)	Prospective, double-blind, randomised, placebo controlled trial	Participants received either GOS/FOS prebiotic supplement in the intervention group (n = 66) or maltodexitrin supplementation in the controll group (n = 68)	Cumulative incidences for AD, recurrent wheezing, and allergic urticaria were higher in the placebo group after 2 years
4	Grüber et al.—2010, 2016 [50,51]	Healthy infants with low risk of atopy (n = 1130)	Double-blind, controlled, randomized, prospective intervention study	Participants were divided into thre groups: prebiotic group (n = 414) -mixture of GOS, FOS, pAOS, breastfed group (n = 300), control group (n = 416)	After 1 year, AD occurred in significantly fewer infants from the prebiotic group
5	Niele et al.—2012 [52]	Preterm infants (n = 94)	Prospective, double-blind, randomised, placebo controlled trial	Volunteers received either enteral GOS, FOS and pAOS supplementation (n = 48) or placebo (n = 46) during first month of life	No decrease in the incidence of allergic and infectious diseases during first year of life
6	Pontes et al.—2016 [53]	Healthy children (1–4 years of age) (n = 256)	Double-blind, randomized, controlled trial	The intervention group (n = 125) received cow’s milk-based beverage containing DHA, PDX, GOS, β-glucan and the control group (n = 131) were fed cow’s milk three servings/day up to 28 weeks	Participants in the intervention group were associated with fewer episodes of allergic manifestations
7	Boyle et al.—2016 [54]	Infants with an atopic parent (n = 1047)	Parallel-group, multicentre, randomized double-blind controlled trial	Three groups: prebiotic group (n = 432) -mixture of GOS, FOS, pAOS, breastfed group (n = 184), control group (n = 431)	Prebiotics did not prevent AD in the first year of life
8	Ranucci et al.—2018 [55]	Infants (n = 400) with an atopic parent	Randomised, double-blind, placebo-controlled trial	Participants received either prebiotic formula containing GOS/PDX (n = 201) or standard formula (n = 199) in the first 48 weeks of life	No significant differences in the cumulative incidence of AD and its intensity and duration between groups
9	Wopereis et al.—2018 [56]	Healthy infants (n = 138)	Double-blind, randomized, controlled parallel-group nutritional intervention trial	Participants were divided into thre groups: prebiotic group (n = 51) -mixture of GOS, FOS, pAOS, breastfed group (n = 30), control group (n = 57)	Metabolites and pH of infants receiving GOS/FOS/pAOS was closer to breastfed infants than to infants receiving standard cow’s milk formula. After 18 months, AD occurred in significantly fewer infants in the prebiotic group

Abbreviations: AD, atopic dermatitis; FOS, fructo-oligosaccharides; GOS, galacto-oligosaccharides; LOS, lactulose; pAOS, acidic oligosaccharides; PDX, polydextrose.

**Table 3:** Pre- and probiotics in the treatment of AD in infants.

No. of Study	Author	Patient Population (Number)	Type of Study	Intervention	Results
1	Majamaa et al.—1997 [57]	Infants aged 2.5 to 15.7 months with AD (n = 27), nursing mothers of infants with AD (n = 10)	Randomized controlled trial	Infants with AD and cow’s milk allergy received cow’s milk without (n = 14) and with (n = 13) the addition of LGG; the second part of the study involved 10 breast-fed infants who had AD and cow’s milk allergy. In this group LGG was given to nursing mothers	Probiotic bacteria downregulated hypersensitivity reactions and intestinal inflammation
2	Isolauri et al.—2000 [58]	Infants with AD, mean age of 4.6 months (n = 27)	Randomized double-blind placebo-controlled study	Probiotic-supplemented, Bb-12 (n = 9) or LGG ATCC 53103 (n = 9), extensively hydrolysed whey formulas or to the same formula without probiotics (n = 9)	First clinical demonstration of specific probiotic strains modifying AD
3	Kirjavainen et al.—2003 [59]	Infants with AD, mean age was 5.5 months (n = 43)	Randomized double-blind manner	Infants were randomly assigned into placebo (n = 10), viable LGG (n = 17), or heat-inactivated LGG groups (n = 16) and extensively hydrolyzed whey formula or the same formula supplemented with viable or heat-inactivated LGG	Supplementation of infant formulas with viable but not heat-inactivated probiotic was effective for the management of AD and cow’s milk allergy
4	Viljanen et al.—2005 [60]	Infants with AD under the age of 12 months (n = 230)	Randomized double-blinded study	First group (n = 80) received capsules containing LGG ATCC 53103; the second group (n = 76) a mixture of probiotics: LGG, *L. rhamnosus* LC705, *B. breve* Bbi99, and *Propionibacterium* JS; and there was a placebo group (n = 74)	Treatment with *L. rhamnosus* alleviated AD symptoms in IgE-sensitized infants
5	Weston et al.—2005 [61]	Children aged 6-18 months with moderate or severe AD (n = 56)	Randomised double blind placebo controlled trial	The children were given a *L. fermentum* VRI-033 PCC (n = 28) or placebo (n = 28), twice daily for 8 weeks	Supplementation with probiotic bacteria is beneficial in improving the extent and severity of the symptoms
6	Taniuchi et al.—2005 [62]	Infants with cow milk hypersensitivity and AD (n = 10)	Randomised placebo controlled trial	Orally given lyophilized bifidobacteria *B. breve* M-16V (n = 10) strain or placebo (n = 7)	Significantly increased proportion of bifidobacteria in the fecal microflora
7	Folster-Holst et al.—2006 [63]	Infants (n = 54) aged 1-55 months with moderate-to-severe AD	Randomized, double-blind, placebo-controlled study	LGG (n = 26) or placebo (n = 27) was received during an 8-week period	No significant differences between the groups in the clinical symptoms
8	Brouwer et al.—2006 [64]	Infants less than 5 months old with AD (n = 50)	Randomized, double-blind, placebo-controlled study	Participants received a hydrolysed whey-based formula as placebo (n = 17), or supplemented with either *L. rhamnosus* (n = 17) or LGG (n = 16) for 3 months	No clinical or immunological effect of *L. rhamnosus*
9	Grüber et al.—2007 [65]	Infants with AD aged 3–12 months (n = 54)	Randomized trial	LGG (54) or placebo (48) as a food supplement for 12 weeks	No therapeutic effect of probiotic against mild to moderate AD
10	Flintermann et al.—2007 [66]	Children aged 0.5–2.8 years with AD (n = 13)	Randomized trial	Probiotics (n = 7) or placebo (n = 6) was randomly assigned to the patients. The probiotics contained a mixture of *L. acidophilus* W55, *L. casei* W56, *L. salivarius* W57, *L. lactis* W58, *B. infantis* W52, *B. lactis* W18 and *B. longum* W51	Probiotics enhanced the production of Th1 and regulatory cytokines *in vitro*
11	van der Aa LB et al.—2010 [67]	Infants with AD SCORAD > or =15, aged < 7 months and exclusively formula fed (n = 90)	Double-blind, placebo-controlled multi-centre trial	Extensively hydrolysed formula with *B. breve* M-16V and a galacto-/fructo-oligosaccharide mixture (n = 46) or the same formula without synbiotics (n = 44) for 12 weeks	Synbiotic mixture does not have a beneficial effect on the severity of AD, but it modulates the intestinal microbiota
12	Gøbel et al.—2010 [68]	Children from 7 to 24 months of age with AD (n = 50)	Randomised double-blind placebo-controlled trial	First group: *L. acidophilus* NCFM and other supplements in a capsule given (n = 17), Second group: *B. lactis* Bi-07 and supplements in a capsule given (n = 17). Third group received placebo (n = 16). Treatment was given daily for 8 weeks	No overall beneficial effects on the degree of SCORAD index.
13	Nermes et al.—2011 [69]	Infants with AD (n = 39)	Double-blind study	Extensively hydrolysed casein formula supplemented with (n = 19) or without (n = 20) LGG (ATCC 53103) was given to the two differenct groups for three months	Probiotics may enhance gut barrier function
14	Farid et al.—2011 [70]	Infants and children aged 3 months to 6 years with AD (n = 40)	Randomized, double-blind, placebo-controlled study	Patients in the probiotic group (n = 19) received synbiotic containing a mixture of *L. casei, L. rhamnosus, Str. thermophilus, B. breve, L. acidophilus, B. infantis, Lactobacillus bulgaricus* and FOS twice daily for 8 weeks	Mixture of probiotics and FOS improved the severity of symptoms
15	Gore et al.—2012 [71]	Infants with AD (n = 208)	Randomized-controlled trial	Infants were randomized to daily supplements containing *L. paracasei* or *B. lactis* (n = 137) or placebo (n = 71) for a 3-month period, while receiving extensively hydrolysed whey-formula (dairy-free diet)	No benefit in the treatment of eczema and no effect on the progression of allergic disease
16	Shafiei et al.—2011 [72]	Infants aged 1-36 months with moderate-to-severe AD (n = 41)	Randomized double blind-placebo controlled trial	Mixture of seven strain probiotics plus FOS (n = 20) or placebo (n = 21), administered daily as a powder for two months	No improvement of AD
17	Ivakhnenko et al.—2013 [73]	Infants aged of 3-12 months with the diagnosis of AD and allergy to cow’s milk protein (n = 60)	Open randomized prospective clinical study	Bb-12 and *Str. thermophilus* TH-4 intake for half of volunteers (n = 30). The other half of the volunteers (n = 30) received placebo for 4 weeks	Improved clinical symptoms
18	Lin et al.—2015 [74]	Infants with AD (n = 40)	Randomized controled study	The intervention group (n = 20) received *B. bifidum* triple viable capsules for 4 weeks with a dosage of one capsule three times a day. The control group (n = 20) were not given a placebo drug	Positive effect on the prevention and treatment
19	Guo et al.—2015 [75]	Adult AD patients (n = 180)	Randomized trial	Participants were divided into two groups. Participants received routine symptomatic treatment and combination of probiotics (microecologics) (n = 90) or symptomatic treatment (n = 90) orally twice a day for one month	Application of microecologics as an adjuvant therapy was effective
20	Wu et al.—2017 [76]	Children aged 4-48 months with AD and with SCORAD ≥ 15 at enrollment. (n = 66)	Two-center, double-blinded, randomized and placebo-controlled study	Treatment group (n = 33)—one capsule containing *L. rhamnosus* a day, control group (n = 33)—one capsule of placebo a day for 8 weeks	Probiotic was effective in decreasing AD symptoms

Abbreviations: AD, atopic dermatitis; *B., Bifidobacterium*; Bb-12, *Bifidobacterium lactis* Bb-12; FOS, fructo-oligosaccharides; IFNγ, Interferon gamma; IL-10, Interleukin 10; *L., Lactobacillus*; LGG, *Lactobacillus rhamnosus* GG; *Propionibacterium* JS, *Propionibacterium freudenreichii* ssp. *shermanii* JS; SCORAD, Scoring Atopic Dermatitis; *Str.: Streptococcus*, Th1, T helper type 1 cells.

**Table 4:** Pre- and probiotics in the treatment of AD in children.

No. of Study	Author	Patient Population (Number)	Type of Study	Intervention	Results
1	Rosenfeldt et al.—2003 [77]	Children aged 1 to 13 years with AD (n = 43)	Double-blind, placebo-controlled, crossover study	The patients were randomized in two groups to receive either placebo followed by active treatment or active treatment followed by placebo. 2 probiotic lyophilized *L. rhamnosus* 19070-2 and *L. reuteri* DSM 122460 were given in combination for 6 weeks	Combination of probiotics was significantly effective in the management of AD
2	Sistek et al.—2006 [78]	Children aged between 1 and 10 years with AD (n = 59)	Randomized controlled trial	*L. rhamnosus* and *B. lactis* (n = 29) or placebo (n = 30) were given daily as a powder for 12 weeks	Combination of probiotic bacteria improved AD only in food sensitized children
3	Passeron et al.—2006 [79]	Children aged at least 2 years with AD (n = 48)	Double-blind prospective randomized study	*L. rhamnosus* Lcr35 plus prebiotic preparation (n = 24) or prebiotic preparation alone (n = 24) was given three times a day for 3 months	Both synbiotics and prebiotics used alone seem able to significantly improve the manifestations of AD
4	Gerasimov et al.—2010 [80]	Children aged 1–3 years with moderate-to-severe AD (n = 90)	Randomized, double-blind, placebo-controlled, prospective trial	Infants were randomly assigned into placebo (n = 47), and intervention group (n = 43). Mixture of *L. acidophilus* DDS-1, *B. lactis* UABLA-12 with fructo-oligosaccharide or placebo twice daily for 8 weeks	Significant clinical improvement
5	Woo et al. —2010 [81]	Children aged 2 to 10 years with AD (n = 45)	Double-blind, placebo-controlled trial	Volunteers received either *L. sakei* KCTC 10755BP (n = 45) or placebo (n = 33) daily for 12 weeks	Substantial clinical improvement and a significant decrease in chemokine levels
6	Han et al.—2012 [82]	Children aged 1–13 years presenting with AD (n = 83)	Randomized, double-blind, placebo-controlled study	*L. plantarum* CJLP133(n = 44) or placebo (n = 39) was given to children twice a day for 12 weeks. SCORAD scores, eosinophil counts, serum total IgE, IFN-γ and IL-4 were evaluated	SCORAD score at week 14 was significantly lower in the probiotic group
7	Wu et al.—2012 [83]	Children aged 2-14 years with moderate-to-severe AD (n = 54)	Double-blind, randomized, clinical trial	One capsule twice daily for 8 weeks containing either *L. salivarius* and FOS (n = 27) or FOS only (n = 27)	Synbiotic combination was superior to the prebiotic alone
8	Yesilova et al.—2012 [84]	Children suffering from a moderate-to-severe AD, 1-13 years of age (n = 40)	Double-blind, randomized, placebo-controlled study	The probiotic group (n = 20) was administered with a probiotic complex containing *B. bifidum, L. acidophilus, L. casei,* and *L. salivarius* for 8 weeks. The placebo group (n = 20) was administered skim milk powder and dextrose	Probiotics to be effective in reducing SCORAD index, serum IL-5, IL-6, IFN-γ, and total serum IgE levels but not effective in reducing serum IL-2, IL-4, IL-10, ECP, or TNF-α levels
9	Yang et al.— 2014 [85]	Children aged 2-9 years with AD (n = 100)	Randomized, double-blind, placebo-controlled, parallel trial	Randomly allocated to the probiotics (*L. casei, L. rhamnosus, L. plantarum, B. lactis*) (n = 50) or placebo (n = 50) groups for 6 weeks	Probiotics successfully colonized in the intestine; but additional effects were not found
10	Wang et al.—2015 [86]	Children aged 1-18 years with moderate-to-severe AD (n = 210)	Double-blind, prospective, randomized placebo-controlled study	The groups received *L. paracasei* (n = 55) or *L. fermentum* (n = 55) or *L. paracasei* and *L. fermentum* mixture (n = 55) or placebo (n = 55) for 3 months	Supplementation of a probiotic mixture was associated with clinical improvement

Abbreviations: AD, atopic dermatitis; *B., Bifidobacterium*; ECP, Eosinophil cationic protein; FOS, fructo-oligosaccharides; IFNγ, Interferon gamma; IgE, immunoglobulin E; IL-2, interleukin 2; IL-4, interleukin 4; IL-5, interleukin 5; IL-6, interleukin 6; IL-10, interleukin 10; *L., Lactobacillus*; SCORAD, Scoring Atopic Dermatitis; TNF-α, tumor necrosis factor alpha.

**Table 5:** Pre- and probiotics in the treatment of AD in adults.

No. of Study	Author	Patient Population (Number)	Type of Study	Intervention	Results
1	Roessler et al.—2008 [87]	Adults with AD (n = 15) and healthy adults (n = 15)	Double-blind, placebo-controlled, randomized cross-over study	Probiotic containing a combination of probiotics *L. paracasei* Lpc-37, *L. acidophilus* 74-2 and *B. lactis* DGCC 420 in healthy volunteers (n = 15) and in patients with AD (n = 15) given over 8 weeks	Probiotic bacteria transiently colonized the intestines
2	Yoshida et al.—2010 [88]	Adults with AD (n = 24)	Randomized, placebo-controlled study	Intervention group (n = 16) were given either *B. breve* strain YY or patients received placebo (n = 8) for 8 weeks	Probiotic was beneficial for the treatment of AD
3	Drago et al.—2012 [89]	Adult patients between 18 and 46 years with moderate-to-severe AD (n = 38)	Parallel-group double-blind placebo-controlled randomised trial	Clinical efficacy of the intake of *L. salivarius* LS01 (n = 19) in the treatment of adult patients with AD	Positively modified clinical and immunologic status and life quality
4	Iemoli et al.—2012 [90]	Adult AD patients (n = 48)	Randomized double-blinded active treatment versus placebo study	Intake of a combination of two probiotics: *L*. *salivarius* LS01 and *B. breve* BR03 for 12 weeks in the probiotic group (n = 16)	Beneficial effects for clinical and immunologic alterations
5	Matsumoto et al. [91]	Adult patients with AD (n = 44)	Randnomized controlled trial	Patients were randomly assigned to receive LKM512 (n = 22) or a placebo (n = 22)	LKM512 exerted antipruritic effects by increasing kynurenic acid production
6	Drago et al.—2014 [92]	Adult patients with AD (n = 25)	Prospective, controlled pilot trial	*L. salivarius*, *Str. thermophilus* ST10 and tara gum intake for half of participants (n = 13). The other half of the participants (n = 12) received placebo for 1 month	The combination of tara gum and probiotics increases the efficacy of other probiotic strains
7	Nakatsuji et al.—2021 [93]	Adult patients with AD (n = 54)	Double-blinded, randomized trial	1-week trial of topical *Staphylococcus hominis* A9 (ShA9) or vehicle on the forearm skin of 54 adults with *S. aureus*-positive AD	Participants receiving ShA9 had fewer adverse events associated with AD; eczema severity was not significantly different when evaluated in all participants treated with ShA9 but a significant decrease in S. aureus and increased ShA9 DNA were seen

Abbreviations: AD, atopic dermatitis; *B., Bifidobacterium*; *L., Lactobacillus*; LKM512, *Bifidobacterium animalis* subsp. *lactis* LKM512; *S., Staphylococcus*; *Str., Streptococcus*.

**Table 6:** Probiotic application in psoriasis.

No. of Study	Author	Patient Population (Number)	Type of Study	Intervention	Results
**HUMAN MODEL—PROBIOTICS ADMINISTERED ORALLY**					
1	Vijayashankar, Raghunath.—2012 [94]	A patient with generalised pustular psoriasis (n = 1)	Case report	*L. sporogene*, one sachet thrice daily	In 15 days, the fever subsided, lesions started involuting and no new lesions appeared
2	Groeger et al.—2013 [95]	Patients with psoriasis (n = 26), patients with ulcerative colitis and chronic fatigue syndrome (n = 70), healthy volunteers (n = 35)	Randomized, double-blind, placebo-controlled	Sachets containing *B. infantis* 35264 (n = 63) or placebo containing maltodextran (n = 55) daily for 8 weeks	Significant decrease in CRP and TNF-α levels
3	Navarro-Lopez et al.— 2019 [96]	18–70 year old adults withplaque psoriasis (n = 90)	Randomized, double-blind, placebo-controlled	Participant were randomized into probiotic (n = 45) and placebo (n = 45) groups. In the probiotic group capsule containing a mixture of 3 probiotic strains in 1:1:1 ratio (*B. longum* CECT 7347, *B. lactis* CECT 8145 and *L. rhamnosus* CECT 8361) was given for 12 weeks	Lower risk of relapse following the administration of probiotic bacteria, which reduced PASI75 in 66.7% of the patients. In the placebo group, 41.9% of patients showed reduction. In PGA index 48.9% of the probiotic group reached a score of 0 or 1 compared to 30.2% in the placebo group
**ANIMAL MODEL—PROBIOTICS ADMINISTERED TOPICALLY AND ORALLY**					
4	Chen et al.—2017 [97]	Male BALB/c; imiquimod-induced epidermal hyperplasia and psoriasis-like skin inflammation (n = 24)	Animal study	In the intervetion group mice were fed orally with differentdoses of *L. pentosus* GMNL-77 or with the vehicle control (distilled water) for 7 consecutive days	Improvement of skin symptoms, decreased TNF-α, IL-6, IL-23, IL-17A/F, and IL-22 levels in the skin, and reduced number of IL-17- and IL-22-producing CD4+ T cells
5	Rather et al.—2018 [98]	Mice with imiquimod-induced psoriasis-like skin inflammation (n = 30)	Animal study	Mice divided into five different groups, 6 mice each: control group, imiquimod group, imiquimod+vaseline group, imiquimod+clobetasol group, and imiquimod+ ethanolic extract of *L. sakei* Probio65	Significant inhibition of imiquimod-induced skin inflammation
6	Lu et al.— 2021 [99]	Female BALB/c mice (n = 60)	Animal study	Mice were separated into 10 groups (6 included in each group): control group, imiquimod group, methotrexate positive control group and probiotic groups (seven groups); CCFM667 *B. adolescentis*, CCFM1078 *B. breve*, CCFM1148 *B. animalis*, CCFM1147 and CCFM1074 *L. paracasei*, CCFM1032 and CCFM1040 *L. reuteri*	Four probiotic bacteria groups ameliorated psoriasis-like pathological characteristics and suppressed the release of IL-23/T helper cell 17 axis-related inflammatory cytokines
7	Ogawa C. et al.—2021 [100]	Mice with imiquimod-induced psoriasis	Animal study	Mice were administered *L. mesenteroides* for 21 days alongside the topical application of imiquimod on the dorsal skin for 6 consecutive days	Suppressed erythema, scaling, upregulated IL-17 production, increased levels of plasma deoxycholic acid, altered the faecal microbiota composition

Abbreviations: *B.*, *Bifidobacterium*; CRP, C-reactive protein; IL-6, interleukin 6; IL-17, interleukin 17; *L., Lactobacillus; L. mesenteroides, Leuconostoc mesenteroides; L. reuteri, Limosilactobacillus reuteri;* PASI75, Psoriasis Area Severity Index 75%; PGA, Physician Global Assessment.

**Table 7:** Probiotic application in chronic ulcers.

No. of Study	Author	Patients (Number)	Type of Study	Intervention	Results
**HUMAN MODEL—PROBIOTIC SUPPLEMENTATION**					
1	Peral et al.—2010 [101]	Patients aged 40–70 years of age; patients suffered from type 2 diabetes mellitus (n = 14); non-diabetic (n = 20); inclusion criteria: venous ulcer; infection and no signs of healing in the past 3 months, despite conventional medical treatment	Interventional study	Wounds were treated with topical applications of a whole culture of *L. plantarum* ATCC; the culture was applied once-daily over a period of 10 days	After 30 days of treatment, a reduction of more than 90% of the wound area was observed in 43% and 50% of the diabetic and non-diabetic patients, respectively
2	Mohseni et al.—2018 [102]	Patients aged 40-85 years old with grade 3 diabetic foot ulcer (n = 60)	Randomized, double-blind, placebo-controlled trial	Participants were randomly divided into two groups (n = 30/group) to receive either probiotic or placebo daily for 12 weeks. The probiotic mix consisted of *L. acidophilus, L. casei, L. fermentum, B. bifidum*	Beneficial effects on ulcer size, glycaemic control, total cholesterol, CRP, plasma nitric oxide, total antioxidant capacity and malondialdehyde levels
3	Venosi et al.—2019 [103]	83-year-old woman with a critical limb ischemia and an infected difficult-to-treat ulcerated cutaneous lesion of the right leg	Case report	Mixture of probiotic bacteria (lyophilized powder sachets, containing *Lactobacillus plantarum*, *Lactobacillus acidophilus* and *Str. thermophilus)* against different bacteria species *K. pneumonia, P. mirabilis and E. faecalis*	Treatment was effective against the three bacteria species
**ANIMAL MODEL—PROBIOTICS SUPPLEMENTATION AND TOPICAL APPLICATION**					
4	Jones et al.—2012 [104]	New Zealand white rabbit (n = 4)	Animal study	The wounds were treated with control or gNO-producing patches designed to produce gNO levels. Wounds are not infected (1. and 2. rabbit) or infected (3. and 4. rabbit). Wounds are treated with placebo (1. and 3. rabbit) or with gNO producing patches (2. and 4. rabbit)	Histological analysis showed improved wound healing in gNO-producing patch-treated rabbits
5	Stefia et al.—2020 [105]	C57BL/6 wild type wounded mice (n = 30)	Randomized controlled trial in mice	Mice were wounded and divided into 3 groups (n = 10/group); receiving topical applications Pluronic gel containing either vehicle alone or the supernatant fractions prepared from *F. prausnitzii* strains A2-165 or AHMP21	Probiotic can regulate wound inflammation and accelerate wound closure
6	Kusumaningsih et al.—2021 [106]	Male Wistar rats (n = 36)	Animal study	Rats were wounded and divided intor 6 groups (n = 6/group); (1) a control group over 3 days, (2) a group that used distilled water over 7 days, (3) a group that underwent topical treatment over 3 days, (4) a group that used probiotic (*L. casei*) administered topically over 7 days, (5) a group that underwent systemic treatment over 3 days (6) a group that took oral probiotics for the traumatic ulcers over 7 days	Significant differences were observed in the number of fibroblasts and blood vessels
**IN VITRO STUDIES—PROBIOTICS APPLICATION**					
7	Vågesjö et al.—2018 [107]	Human skin wound model/mice	In vitro model of wound reepithelialization	Wounds were treated daily with saline solution, control *Lactobacillus reuteri* or CXCL12-expressing *L. reuteri* or *L. lacti*	Promising therapeutic approach for non-healing wounds
8	Coman et al.—2020 [108]	Pathogenic bacteria were isolated from chronic ulcerative lesions	In vitro study	To evaluate probiotic efficacy of SYNBIO (1:1 combination of *L. rhamnosus IMC 501* and *L paracasei IMC 502*) in wound infections	Good antimicrobial capacity and adhesion percentage to human keratinocyte cells and fibroblasts

Abbreviations: *B., Bifidobacterium*; CRP, C-reactice protein; *E., Enterococcus*; *F., Faecalibacterium*; gaseous nitric oxide, gNO; *K., Klebsiella; L.*, *Lactobacillus; P.*, *Proteus; Str., Streptococcus*.

**Table 8:** Probiotic treatment in SD.

No. of Study	Author	Patient Population (Number)	Type of Study	Intervention	Results
1	Guéniche et al.—2008 [109]	Volunteers aged 6 to 70 years suffering from SD (n = 60)	Prospective, double-blind, placebo-controlled	A cream containing a 5% lysate of the nonpathogenic bacteria *V. filiformis* (n = 30) or a vehicle cream applied once daily for 4 weeks (n = 30)	Significant improvement of SD
2	Reygagne et al.—2017 [110]	Male volunteers aged 18 to 60 years with moderate-to-severe dandruff (n = 60)	Randomized, placebo-controlled study	A sachet containing ST11 (n = 30) or a placebo (n = 30) administered orally for 56 days	Significantly reduced severity of dandruff

Abbreviations: SD, seborrheic dermatitis; ST11, *Lactobacillus paracasei* ST11; *V. filiformis*, *Vitreoscilla filiformis*.

**Table 9:** Probiotic application of burns in humans and animal models.

No. of Study	Author	Patient Population (Number)	Type of Study	Intervention	Results
**HUMAN MODEL—PROBIOTICS ADMINISTERED ORALLY OR TOPICALLY**					
1	Peral et al.—2009 [111]	Patients with second and third-degree burns (n = 80)	Case-control study	Patients were separated into 2 groups: in the topical probiotic group patients (n = 38) received *L. plantarum* ATCC 10241.In the control group patients (n = 42) received 1% SD-Ag cream for 10 days	Topical probiotic treatment of 2nd degree burn patients was as effective as SD-Ag decreasing pathogen load
2	Stefanatou et al.—[112]	34-year-old woman suffering from extensive deep-partial and full thickness thermal burns	Case report	*S. boulardii* administered for nearly 2 months	Probiotic sepsis due to fungaemia in a critically ill burn patient which resulted in death
3	Thomson et al.—2012 [113]	47-year-old lady with 54% deep-dermal and full-thickness flame burns to her neck, chest, upper abdomen and upper limbs	Case report	Oral administration of *L. casei shirota* for 2 weeks after infection which occurred 5 months after burn	Pathogen of the wound changed from multidrug resistant to multidrug sensitive strain
4	Mayes T et al.—2015 [114]	Less than 22 years old acutely burned patients, and were admitted/consented within 10 days of burn injury (n = 20)	Randomized, double-blind, placebo-controlled	The treatment group received LGG (n = 10). The control group received placebo (n = 10). Investigational products were administered via nasoduodenal feeding tube twice daily	Improved gastrointestinal outcomes and reduced time to wound healing
5	El-Ghazely et al. —2016 [115]	Thermally-injured pediatric patients with total body surface burns between 20-50% and depth between 5-10% (n = 40)	Randomized, double-blinded, controlled trial	Participants were separated into 2 groups; probiotic group (n = 20) received probiotic preparations of *L. fermentum* and *L*. *delbrueckii* and placebo control group (n = 20)	Decreased infection incidence in the probiotic group
6	Perdanakusuma et al.—2019 [116]	Burn patients (n = 16)	Randomized, placebo-controlled trial	Patients were randomized into three treatment groups. Oral administration of either a placebo, a *L. reuteri* probiotic, or a *B. infantis 35624* probiotic for 14 consecutive days	*B. infantis 35624* single-strain probiotic was not significantly superior to *L. reuteri protectis* in altering intestinal immunity after burns
7	Fleming et al.—2019 [117]	Burn patients aged 18 to 89, who were hospitalized for at least 2 weeks, no formal protocol of antibiotics use was estabilished (n = 108)	Retrospective study	Oral administration of >1 million colony-forming units per day of *L. acidophilus* and *L. rhamnosus*	No improvements in patient outcomes but increased incidence of diarrhea
**ANIMAL MODEL—PROBIOTICS ADMINISTERED ORALLY**					
8	Herek et al.—2004 [118]	Male albino rats (n = 23)	Animal study	The rats were divided into sham burn group (n = 7), burn + Ampicillin-sulbactam group (n = 8), burn + Ampicillin-sulbactam + probiotic (*S. boulardii*) group (n = 8) administered twice daily for 5 days	Decreased incidence of antibiotic-induced bacterial translocation
9	Gong et al.—2017 [119]	Healthy male Wistar rats (n = 60)	Animal study	The rats were divided into groups: burn model group (n = 15)—normal saline; glutamine treatment group (n = 15)—glutamine + normal saline; probiotics treatment group (n = 15)—probiotic + normal saline; glutamine and probiotics combined treatment group (n = 15)—glutamine + normal saline. All were administered once daily for 7 days	Glutamine and probiotics together significantly inhibited nitric oxide (NO) content and reduced levels of the inflammatory factors
**ANIMAL MODEL—PROBIOTICS ADMINISTERED TOPICALLY OR LOCALLY**					
10	Valdez et al.—2005 [120]	Adult inbred BALB/c mice	Animal study	*L. plantarum ATCC 10241* injection into burned area on 3, 4, 5, 7 and 9 days	Samples from skin, liver and spleen taken after 5, 10 and 15 days demonstrated inhibition of *P. aeruginosa* colonisation
11	Brachkova et al.—2011 [121]	Male Wistar rats (n = 25)	Animal study	Rats were randomly allocated into groups: non-burned control rats (n = 2); burned control rats (n = 6); burned skin covered with films containing *L. plantarum* (n = 3); burned skin on to with a suspension of *P. aeruginosa* (n = 7); burned skin, contaminated with *P. aeruginosa*, and covered with films containing *L. plantarum* ATCC 8014(7)	Reduction of pathogen load
12	Argenta et al. —2016 [122]	Female C57 BL/b mice (n = 38)	Animal study	The mice were divided into groups. Injured sites were treated with vehicle (burn wound control), probiotics (*L. plantarum* ATCC 1024) only, pathogenic bacteria (*P. aeruginosa*) only, or probiotics + pathogen (*Lactobacillus* and *P. aeruginosa*) for five days	Lower mortality rate and inhibition of pathogenic bacteria
13	Satish et al.—2017 [123]	Male Dutch Belted rabbits	Animal study	Each rabbit had four burn wounds created on its dorsum-the four burn wound conditions therefore were: (1) Burn wound only; (2) *L. plantarum* ATCC 10241 only; (3) *P. aeruginosa* only; (4) *L. plantarum + P. aeruginosa*	Curtailed severity and length of infection, reduced scarring
14	Sürmeli et al.—2019 [124]	Rats (n = 35)	Animal study	Rats were divided into groups (n = 7/group): control group; *L. plantarum* applied immediately after the burn and then MRSA inoculated; MRSA applied immediately after the burn and then *L. plantarum* inoculated; control of *L. plantarum*; control of MRSA	Probiotic showed protective role in non-infected burn wounds
15	Khan et al.—2019 [125]	Male BALB/c mice (n = 30)	Animal study	The mice were randomized into negative (untreated), positive (silver sulfadiazine cream), vehicle (biodispersion and nanoscaffold), and experimental bioscaffold groups (n = 6/group). Treatments were applied locally on 2, 6, 10, and 14 days postburn–application of probiotic (*E. mundtii* QAUEM2808)	Accelerated epithelialization, collagen deposition, and hair follicle formation and inhibit pathogens
**HUMAN MODEL–PREBIOTICS OR PREBIOTICS and PROBIOTICS ADMINISTERED ORALLY**					
16	Olguin et al.—2005 [126]	Burn patients (n = 21)	Randomized, double-blind, placebo-controlled	6 g of oligofructose (study group) or sucrose as placebo (control group) during 15 days	No effect on gastrointestinal permeability

Abbreviations: *B., Bifidobacterium*; *E., Enterococcus*; *L., Lactobacillus*; LGG, *Lactobacillus rhamnosus* GG; MRSA, Methicillin-resistant *Staphylococcus aureus*; *P., Pseudomonas*; S., Saccharomyces; SD-Ag, Silver sulphadiazine.

**Table 10:** Pre- and probiotic treatment of acne in humans, animal models and in vitro studies.

No. of Study	Author	Patient Population (Number)	Type of Study	Intervention	Results
**HUMAN MODEL–PROBIOTICS OR PREBIOTICS ORAL SUPPLEMENTATION**					
1	Kim et al.—2010 [35]	Patients with acne (n = 36)	Randomized, double-blind, placebo-controlled study	Fermented milk with lactoferrin daily (n = 18) or fermented milk only (n = 18) for 12 weeks supplemented orally	Improvement of acne with a selective decrease of triacylglycerols in skin surface lipids
2	Fabroccini et al.—2016 [127]	Patients with acne (n = 20)	Placebo–controlled trial	Over a 12-week period, the probiotic group (n = 10) consumed a liquid supplement containing LSP1, placebo group (n = 10)	Normalised skin expression of genes involved in insulin signalling and improvement of acne
3	Dall’Oglio et al.—2018 [128]	Female patients with mild to moderate acne (n = 12)	Proof of concept pilot trial	Prebiotic oral supplementation with food supplement containing FOS and GOS for 3 months	Positive effects on glycemic and lipid metabolic parameters
4	Rahmayani et al.—2019 [129]	Patients with acne aged between 17 and 25 years old (n = 33)	Pre-experimental clinical study with a pretest-posttest design	Oral mix of probiotics was given to individuals for 30 days-*B. lactis* W51, *B. lactis* W52, *L. acidophilus* W55, *L.* *casei* W56, *L.* W57, *L. lactis* W58	Elevated serum IL-10 levels
**HUMAN MODEL–PREBIOBIOTICS OR PREBIOTICS TOPICAL APPLICATION**					
5	Kang et al.—2009 [130]	12 years of age or older patients with acne (n = 70)	Double-blind, randomized, placebo-controlled trial	*E. faecalis SL-5* lotion (n = 35) or placebo lotion (n = 35) to apply twice a day for 8 weeks	Reduced number of inflammatory lesions
6	AOBiome LLC.—2019 [131]	Adult patients with mild to moderate acne (n = 358)	Double-blind, randomized, placebo-controlled trial	Probiotic (*N. eutropha*) or placebo spray to saturate the entire face in the morning and at night for 12 weeks	2-point reduction in IGA of acne severity compared to vehicle control
7	De Los Angeles Mosquera Tayupanta et al.—2019 [132]	Patients from 15 to 20 years old, with previous diagnosis of type II acne (n = 20)	Interventional study	First the evaluation of the in vitro antagonistic effect of *L. acidophilus* against *C. acnes* was performed, then topical application	Decrease in the population of *C. acnes*
**IN VITRO STUDIES**					
8	Al-Ghazzewi et al.—2010 [133]	-	In vitro study	The synbiotic ability of probiotic bacteria and konjac glucomannan hydrolysates to inhibit acne-inducing bacterium, *C. acnes* growth was studied in vitro	Inhibition of the growth of *C. acnes*, which was significantly enhanced by the presence of prebiotic
9	Kang et al.—2012 [134]	-	In vitro study	Study examined the effects of *L. reuteri* strains (KCTC 3594, KCTC 3678, KCTC 3679) on the proliferation of *C. acnes* and *S. epidermidis*	Control of the growth of bacteria involved in acne inflammation and prevent acne
10	Lee et al.—2012 [135]	-	In vitro study	Activity of *Bifidobacterium spp.* against *C. acnes* KCTC3320 using the co-culture method was investigated	*Bifidobacterium spp*. could be used as an effective treatment and reduced the risk of acne development
11	Khalfallah et al.—2021 [136]	-	In vitro study	Two type of *Str. salivarius* strains and one *L. plantarum* were tested for production of antimicrobials-target organisms used were *C. acnes, S. aureus,* and *P. aeruginosa*	Probiotic containing could be topically applied without the need for a regular antibiotic treatment or as an adjunctive therapy

Abbreviations: B., *Bifidobacterium*; BMI, body mass index; *C., Cutibacterium; E., Enterococcus*; FOS, fructo-oligosaccharides; GOS, galacto-oligosaccharides; IL-10, interleukin 10; LSP1, *Lactobacillus rhamnosus SP1*; *L., Lactobacillus; N., Nitrosomonas; S., Staphylococcus; Str., Streptococcus; P., Pseudomonas; spp.,* species.
